# Supplementary material for: miRNA Clusters with Down-Regulated Expression in Human Colorectal Cancer and Their Regulation
Source: Int J Mol Sci. 2020 Jun 29;21(13):4633. doi: 10.3390/ijms21134633 (PMC7369991; doi:10.3390/ijms21134633)
Supplement: Supplementary file 1 [file ijms-21-04633-s001.zip › S1 Table.docx]

## **S1 Table:** Affiliation of miRNAs to corresponding family based on seed sequence

| **miRNA** | **seed sequence** ^181^ | **family** | **cluster** |
| --- | --- | --- | --- |
| let-7a-5p | GAGGUAG | let-7/98/4458/4500 | miR-100/let-7a-2/miR-125b-1 |
| let-7c-5p | GAGGUAG | let-7/98/4458/4500 | miR-99a/let-7c |
| let-7e-5p | GAGGUAG | let-7/98/4458/4500 | miR-99b/let-7e/miR-125a |
| miR-99a-5p | ACCCGUA | miR-99/100 | miR-99a/let-7c |
| miR-99b-5p | ACCCGUA | miR-99/100 | miR-99b/let-7e/miR-125a |
| miR-100-5p | ACCCGUA | miR-99/100 | miR-100/let-7a-2/miR-125b-1 |
| miR-125a-5p | CCCUGAG | miR-125/4319 | miR-99b/let-7e/miR-125a |
| miR-125b-5p | CCCUGAG | miR-125/4319 | miR-100/let-7a-2/miR-125b-1 |
| miR-1-3p | GGAAUGU | miR-1/206/613 | miR-1-2/133a-1, miR-1-1/133a-2 |
| miR-206-3p | GGAAUGU | miR-1/206/613 | miR-206/133b |
| miR-133a-3p | UUGGUCC | miR-133 | miR-1-2/133a-1, miR-1-1/133a-2 |
| miR-133b-3p | UUGGUCC | miR-133 | miR-206/133b |
| miR-192-5p | UGACCUA | miR-192/215 | miR-192/194-2 |
| miR-215-5p | UGACCUA | miR-192/215 | miR-215/194-1 |
| miR-194-5p | GUAACAG | miR-194 | miR-192/194-2, miR-215/194-1 |
| miR-15a-5p | AGCAGCA | miR-15/16/195/424/497/6838 | miR-15a/16-1 |
| miR-15b-5p | AGCAGCA | miR-15/16/195/424/497/6838 | miR-15b/16-2 |
| miR-16-5p | AGCAGCA | miR-15/16/195/424/497/6838 | miR-15a/16-1, miR-15b/16-2 |
| miR-497-5p | AGCAGCA | miR-15/16/195/424/497/6838 | miR-497/195 |
| miR-195-5p | AGCAGCA | miR-15/16/195/424/497/6838 | miR-497/195 |
| miR-143-3p | GAGAUGA | miR-143/4770/6088 | miR-143/145 |
| miR-145-5p | UCCAGUU | miR-145/5195 | miR-143/145 |
| miR-302a-3p | AAGUGCU | miR-302/372/373/520 | miR-302b/302c/302a/302d/367 |
| miR-302c-3p | AAGUGCU | miR-302/372/373/520 | miR-302b/302c/302a/302d/367 |
