# Supplementary material for: miRNA Clusters with Down-Regulated Expression in Human Colorectal Cancer and Their Regulation
Source: Int J Mol Sci. 2020 Jun 29;21(13):4633. doi: 10.3390/ijms21134633 (PMC7369991; doi:10.3390/ijms21134633)
Supplement: Supplementary file 1 [file ijms-21-04633-s001.zip › S2 Table.docx]

## **S2 Table** Experimental evidence supporting role of miRNAs as tumour suppressors or oncogenes in colorectal cancer (CRC)

| **miRNA** | **levels in CRC** | **correlation of survival with  high expression** | function in CRC  **tumour suppressor** | function in CRC  **oncogene** |
| --- | --- | --- | --- | --- |
| **miR-100/let-7a-2/miR-125b-1, miR-99a/let-7c** and **miR-99b/let-7e/miR-125a** | | | | |
| Let-7a | **down-regulated** ^29,38,39,44,182,183^ | better survival ^184,185^ | ^33,34,38,39,44,182-185^ |  |
| Let-7c | **down-regulated** ^40,86^ | better survival ^186^ | ^40,186^ |  |
| Let-7e | **down-regulated** ^42,44^ |  | ^41,42,44^ | ^187^ |
| miR-99a | **down-regulated** ^65,86^ | better survival ^186^ | ^65,186^ |  |
| miR-99b |  | better survival ^66^ | ^66^ |  |
| miR-100 | **down-regulated** ^29,67,86,188,189^ | better survival ^188^ | ^67,188^ |  |
| miR-125a | **down-regulated** ^23,29,68,69,71,86^ | better survival ^23,69^ | ^23,68,69,71,72^ |  |
| miR-125b | **down-regulated** ^23,189^  **up-regulated** ^75^ | better survival ^186^  worse survival ^73^ | ^186^ | ^73,75^ |
| **miR-1-2/133a-1, miR-1-1/133a-2** and **miR-1-1/133a-2** | | | | |
| miR-1 | **down-regulated** ^76,85-88,190,191^ | better survival ^192^ | ^76,85-89,191^ |  |
| miR-133a | **down-regulated** ^76,95-99, 189,190^ | better survival ^193^ | ^76,78,95-99,193^ |  |
| miR-133b | **down-regulated** ^45,80,100-103,194-196^ | better survival ^194^ | ^45,80,82,100-103,197,198^ |  |
| miR-206 | **down-regulated** ^90,91,93^ | better survival ^90^ | ^77,84,90-94^ |  |
| **miR-192/194-2** and **miR-215/194-1** | | | | |
| miR-192 | **down-regulated** ^57,114,117,119,182^ | better survival ^199^ | ^57,114,119^ |  |
| miR-194 | **down-regulated** ^57,106,113,179^  **without change** ^29^ | better survival ^106,113,199^ | ^105-107,112,113^ | ^104^ |
| miR-215 | **down-regulated** ^57,117,118,182^ | better survival ^118,199,200^  worse survival ^117,201^ | ^57,59,115,116,118-120, 202,203^ |  |
| **miR-15a/16-1** and **miR-15b/16-2** | | | | |
| miR-15a | **down-regulated** ^121,204,205^  **up-regulated** ^206^ | better survival ^204,207^  worse survival ^206^ | ^121,124-127^ | ^208^ |
| miR-15b | **down-regulated** ^136,205^  **up-regulated** ^137,209^ | better survival ^135^  worse survival ^137^ | ^122,135,136^ | ^137^ |
| miR-16 | **down-regulated** ^123,204,205,211^  **up-regulated** ^9,210^ | better survival ^204,207,211^  worse survival ^210^ | ^124,128,130,131^ | ^210^ |
| **miR-143/145** | | | | |
| miR-143 | **down-regulated** ^86,139,155,157,182,212-217^ | better survival ^146,201^ | ^139,153-157,215^ |  |
| miR-145 | **down-regulated** ^86,139,150,152,182, 212-216^ | better survival ^29, 192,199, 218^ | ^62,86,139, 141,143,146,149, 150-152,215^ |  |
| **miR-302b/302c/302a/302d/367** | | | | |
| miR-302a | **down-regulated** ^161^ | better survival ^161^ | ^160-163^ |  |
| miR-302c | **down-regulated** ^164^ | better survival ^164^ | ^24,159,164,165^ |  |
| **miR-497/195** | | | | |
| miR-497 | **down-regulated** ^64,166-172, 205^ | better survival ^166^ | ^166,169-173^ |  |
| miR-195 | **down-regulated** ^169,175,177,178,205,216^ | better survival ^166,205^ | ^166,173,176-178^ | ^180^ |
