# Supplementary material for: miRNA Clusters with Down-Regulated Expression in Human Colorectal Cancer and Their Regulation
Source: Int J Mol Sci. 2020 Jun 29;21(13):4633. doi: 10.3390/ijms21134633 (PMC7369991; doi:10.3390/ijms21134633)
Supplement: Supplementary file 1 [file ijms-21-04633-s001.zip › S3 Table.docx]

## **S3 Table:** miRNA target genes supported by experimental evidence in colorectal cancer (CRC) tissue or cells

| **cluster** | **targets in CRC** |
| --- | --- |
| **miR-100/let-7a-2/miR-125b-1, miR-99a/let-7c** and **miR-99b/let-7e/miR-125a** | |
| **miR-100-5p** | *RAP1B* ^67^ |
| **let-7a-5p** | *MYC* ^33^, *RTKN* ^39^*, UHRF2* ^38^ |
| **let-7a-3p** | *ABCC1* ^34^ |
| **miR-125b** | *APC* ^75^, *MCL1* ^73^ |
| **miR-99a-5p** | *MTOR* ^65^ |
| **let-7c-5p** | *KRAS*, *MMP11*, *PBX3* ^40^ |
| **miR-99b-5p** | *MTOR* ^66^ |
| **let-7e-5p** | *DCLK1* ^41^, *IGF1R* ^43,44^, *ST8SIA1* ^42^ |
| **miR-125a-5p** | *BCL2*, *BCL2L12* ^68^, *CREB5* ^36^, *MCL1* ^68^, *PADI2* ^35^, *SMURF1* ^70^, *TAZ* ^71^, *VEGFA* ^69^ |
| **miR-125a-3p** | *FUT5*, *FUT6* ^72^ |
| **miR-1-2/133a-1, miR-1-1/133a-2** and **miR-1-1/133a-2** | |
| **wh. cluster** | *TAGLN2* ^76^ |
| **miR-1-3p** | *LASP1* ^85^, *HIF1A* ^89^, *NAIP* ^86^, *NOTCH3* ^88^, *VEGF* ^87^ |
| **miR-133a-3p** | *EIF4A1* ^99^, *FSCN1* ^97^, *LASP1* ^96^, *RFFL* ^95^, *SENP1* ^98^ |
| **miR-206-3p** | *BCL2* ^94^, *FMNL2* ^83,91^, *MET* ^91^, *NOTCH3* ^84,92^, *TM4SF1* ^93^ |
| **miR-133b-3p** | *ABCC1* ^197^, *CCN2* ^195^, *CXCR4* ^100^, *DOT1L* ^198^, *EGFR* ^101^, *FTL* ^82^, *HOXA9*^102^, *MET* ^103^, *NUP214* ^45^, *TBPL1* ^196^, *ZEB1*^102^ , *LINC00467* ^82^ |
| **miR-192/194-2** and **miR-215/194-1** | |
| **miR-192-5p** | *BCL2* ^114^, *SRPX2* ^119^, *VEGFA* ^114^, *ZEB2* ^114^ |
| **miR-194-5p** | *AKT2* ^113^, *FOXM1* ^106^, *MAP4K4* ^112^, *THBS1* ^104^, *VAPA* ^105^ |
| **miR-194-3p** | *TGFA* ^107^ |
| **miR-215-5p** | *BMI1* ^59^, *DTL* ^116, 117^, *EREG ^118^*, *HOXB9* ^118^, *SRPX2* ^119^, *TYMS* ^111^, *YY1* ^115^, *ZEB2* ^108,109^ |
| **miR-215-3p** | *CXCR1* ^120^ |
| **miR-15a/16-1** and **miR-15b/16-2** | |
| **miR-15a-5p** | *BCL2* ^125,127^, *BMI1* ^127^, *CCNB1* ^124^, *DCLK1* ^127^, *SOX2* ^125^, *TFAP4* ^126^, *YAP1* ^127^ |
| **miR-15b-5p** | *ACOX1* ^122^, *CHUK* ^136^, *DCLK1* ^135^, *KL ^137^*, *MTSS1* ^137^, *NFKB1* ^136^, *PIM* ^134^ |
| **miR-16-5p** | *CDX2* ^132^, *BIRC* ^131^, *CCNB1* ^124^, *ITGA2* ^130^, *KDR* ^129^*, KRAS* ^128^, *MYB* ^129^, *PTGS2* ^133^, *TFAP4* ^126^ |
| **miR-143/145** | |
| **whole cluster** | *ARF6* ^214^, *IGF1R* ^219^ |
| **miR-143-3p** | *ASAP3* ^156^, *CTNND1* ^155^, *DNMT3A* ^148^, *HK2* ^153^, *ITGA6* ^156^, *KRAS* ^139^, *MAPK7 ^139^*, lncRNA *OECC* ^220^, *PTGS2 ^139^*, *TLR2* ^154^ |
| **miR-143-5p** | *IGF1R* ^157^ |
| **miR-145-5p** | *BAG4* ^141^, *CDK6* ^139^, *CCND2* ^139^, *E2F3* ^139^, *E2F5* ^141^, *ERG* ^152^, *FMNL2* ^141^, *FSCN1* ^149^, *LASP1* ^62^, *MYC* ^139^, *MYO6* ^143^, *NAIP* ^86^, *PXN* ^150^, *ZEB2* ^151^ |
| **miR-302b/302c/302a/302d/367** | |
| **miR-302a-3p** | *NFIB* ^161^ |
| **miR-302c-3p** | *ABCB1* ^165^, *PLAG1* ^24^*, TFAP4* ^164^ |
| **miR-497/195** | |
| **miR-497-5p** | *FOSL1* ^173^, *IGF1R* ^169^, *IRS1* ^170^, *KSR1* ^172^, *PTPN3* ^171^, *VEGFA* ^221^ |
| **miR-195-5p** | *BCL2* ^177^, *CARMA3* ^222^, *CHEK1* ^180^, *FGF2* ^176^, *GDPD5* ^175^, *NOTCH2* ^174,178^, *RBPJ* ^175^, *WEE1* ^180^, *YAP1* ^183^ |
