# Supplementary material for: miRNA Clusters with Down-Regulated Expression in Human Colorectal Cancer and Their Regulation
Source: Int J Mol Sci. 2020 Jun 29;21(13):4633. doi: 10.3390/ijms21134633 (PMC7369991; doi:10.3390/ijms21134633)
Supplement: Supplementary file 1 [file ijms-21-04633-s001.zip › S5 List of abbreviations.docx]

**List of abbreviations**

| ncRNA | non-coding RNA |
| --- | --- |
| miRNA | micro RNA |
| pri-miRNA | primary-miRNA |
| pre-miRNA | precursor-miRNA |
| snoRNA | small nucleolar RNAs |
| lncRNA | long non-coding RNA |
| circRNA | circular RNA |
| lincRNA | long intervening non-coding RNA |

**Abbreviations of ncRNA**

| circRNA PVT1 | Plasmacytoma Variant Translocation 1 locus |
| --- | --- |
| circRNA VAPA | Vesicle-associated membrane protein associated protein A |
| LINC00473 | Long intergenic non-protein coding RNA 473 |
| lincRNA ROR | Large intergenic non-coding RNA Regulator of reprogramming |
| lncRNA ABHD11-AS1 | Abhydrolase domain containing 11 antisense RNA 1 |
| lncRNA ANRIL | Antisense non-coding RNA in the INK4 locus |
| lncRNA CASC15 | Cancer susceptibility candidate 15 |
| lncRNA FTX | Five prime to Xist |
| lncRNA H19 | H19 imprinted maternally expressed transcript |
| lncRNA HOXA11-AS | HOMEOBOX A11 antisense RNA |
| lncRNA LINC00114 | Long intergenic non-protein coding RNA 114 |
| lncRNA LINC00467 | Long intergenic non-protein coding RNA 467 |
| lncRNA LINC00707 | Long intergenic non-protein coding RNA 707 |
| lncRNA MALAT1 | Metastasis associated lung adenocarcinoma transcript 1 |
| lncRNA MEG3 | Maternally expressed 3 |
| lncRNA NEAT1 | Nuclear paraspeckle assembly transcript 1 |
| lncRNA  OECC | Overexpressed in colorectal cancer |
| lncRNA PART-1 | Prostrate androgen-regulated transcript-1 |
| lncRNA SNHG1 | Small nucleolar RNA host gene 1 |
| lncRNA TTN-AS1 | Titin antisense RNA 1 |
| lncRNA UCA1 | Urothelial cancer associated 1 |
| lncRNA UCC | Upregulated in colorectal cancer |
| lncRNA UICLM | Up-regulated in colorectal cancer liver metastasis |
| lncRNA XIST | X inactive specific transcript |
| TP73-AS1 | TP73 antisense RNA 1 |

**Abbreviations of gene names**

| *ABCC1* | ATP binding cassette subfamily C member 1 |
| --- | --- |
| *ABCB1* | ATP binding cassette subfamily B member 1 |
| *ACOX1* | Acyl-coenzyme A oxidase 1 |
| *AKT2* | AKT serine/threonine kinase 2 |
| *AP4* | Activating enhancer binding protein 4 |
| *APC* | APC regulator of WNT signaling pathway |
| *ARF6* | ADP-ribosylation factor 6 |
| *ASAP3* | ArfGAP with SH3 domain, ankyrin repeat and PH domain 3 |
| *BAG4* | BAG cochaperone 4 |
| *BCL2* | BCL2 apoptosis regulator |
| *BCL2L12* | BCL2 like 12 |
| *BIRC* | Survivin |
| *BMI1* | BMI1 proto-oncogene, polycomb ring finger |
| *CARMA3* | Caspase recruitment domain family member 10 |
| *CARMN* | Cardiac mesoderm enhancer-associated non-coding RNA |
| *CCN2* | Cellular communication network factor 2 |
| *CCNB1* | Cyclin B1 |
| *CCND2* | Cyclin D2 |
| *CDK6* | Cyclin-dependent kinase 6 |
| *CDX2* | Caudal type homeobox 2 |
| *CHEK1* | Checkpoint kinase 1 |
| *CREB5* | cAMP responsive element binding protein 5 |
| *CHUK* | Component of inhibitor of nuclear factor kappa B kinase complex |
| *CTNND1* | Catenin delta 1 |
| *CXCR1* | C-X-C motif chemokine receptor 1 |
| *CXCR4* | C-X-C motif chemokine receptor 4 |
| *DCLK1* | Doublecortin like kinase 1 |
| *DLEU2* | Deleted in lymphocytic leukaemia 2 |
| *DNMT3A* | DNA methyltransferase 3 alpha |
| *DOT1L* | DOT1 like histone lysine methyltransferase |
| *DTL* | Denticleless E3 ubiquitin protein ligase homolog |
| *E2F3* | E2F transcription factor 3 |
| *E2F5* | E2F transcription factor 5 |
| *EGFR* | Epidermal growth factor receptor |
| *EIF4A1* | Eukaryotic translation initiation factor 4A1 |
| *ERG* | ETS transcription factor ERG |
| *EREG* | Epiregulin |
| *FSCN1* | Fascin actin-bundling protein 1 |
| *FGF2* | Fibroblast growth factor 2 |
| *FMNL2* | Formin-like 2 protein |
| *FOXM1* | Forkhead box M1 |
| *FOSL1* | FOS like 1, AP-1 transcription factor subunit |
| *FSCN1* | Fascin actin-bundling protein 1 |
| *FTL* | Ferritin light chain |
| *FUT5* | Fucosyltransferase 5 |
| *FUT6* | Fucosyltransferase 6 |
| *GDPD5* | Glycerophosphodiester phosphodiesterase domain containing 5 |
| *IARS2* | Isoleucyl-tRNA synthetase 2, mitochondrial |
| *HK2* | Hexokinase 2 |
| *HIF1A* | Hypoxia inducible factor 1 subunit alpha |
| *HOXA9* | Homeobox A9 |
| *HOXB9* | Homeobox B9 |
| *IGF1R* | Insulin like growth factor 1 receptor |
| *IRS1* | Insulin receptor substrate 1 |
| *ITGA2* | Integrin subunit alpha 2 |
| *KDR* | Kinase insert domain receptor |
| *KL* | Klotho |
| *KRAS* | KRAS proto-oncogene, GTPase |
| *KSR1* | Kinase suppressor of ras 1 |
| *LASP1* | LIM and SH3 protein 1 |
| *MAP4K4* | Mitogen-activated protein kinase kinase kinase kinase 4 |
| *MAPK7* | Mitogen-activated protein kinase 7 |
| *MCL1* | Myeloid cell leukaemia 1 gene |
| *MET* | MET proto-oncogene, receptor tyrosine kinase |
| *MMP11* | Matrix metallopeptidase 11 |
| *MTOR* | Mechanistic target of rapamycin kinase |
| *MTSS1* | MTSS I-BAR domain containing 1 |
| *MYB* | MYC proto-oncogene, bHLH transcription factor |
| *MYO6* | Myosin VI |
| *NAIP* | NLR family apoptosis inhibitory protein |
| *NFIB* | Nuclear factor IB |
| *NFΚB1* | Nuclear factor kappa B subunit 1 |
| *NIRF* | Np95 icbp90 ring finger |
| *NOTCH2* | Notch receptor 2 |
| *NOTCH3* | Notch receptor 3 |
| *NUP214* | Nucleoporin 214 |
| *PADI2* | Peptidyl arginine deiminase 2 |
| *PBX3* | PBX homeobox 3 gene |
| *PIM* | Pim-1 proto-oncogene, serine/threonine kinase |
| *PLAG1* | PLAG1 zinc finger |
| *PTGS2* | Prostaglandin-endoperoxide synthase 2 |
| *PTPN3* | Protein tyrosine phosphatase non-receptor type 3 |
| *PXN* | Paxillin |
| *RAP1B* | RAP1B, member of RAS oncogene family |
| *RBPJ* | Recombination signal binding protein for immunoglobulin kappa J region |
| *RFFL* | Ring finger and FYVE like domain containing E3 ubiquitin protein ligase |
| *RTKN* | Rhotekin |
| *SENP1* | SUMO specific peptidase 1 |
| *SMURF1* | SMAD specific E3 ubiquitin protein ligase 1 |
| *SRPX2* | Sushi repeat containing protein X-linked 2 |
| *ST8SIA1* | ST8 alpha-N-acetyl-neuraminide alpha-2,8-sialyltransferase 1 |
| *SOX2* | SRY-box transcription factor 2 |
| *TAGLN2* | Transgelin 2 |
| *TAZ* | Tafazzin |
| *TBPL1* | TATA-box binding protein like 1 |
| *TFAP4* | Transcription factor AP-4 |
| *TGFA* | Transforming growth factor alpha |
| *THBS1* | Thrombospondin 1 |
| *TLR2* | Toll-like receptor 2 |
| *TM4SF1* | Tetraspanin-like protein called transmembrane 4 L six family member 1 |
| *TYMS* | Thymidylate synthetase |
| *VAPA* | VAMP associated protein A |
| *VEGFA* | Vascular endothelial growth factor A |
| *WEE1* | WEE1 G2 checkpoint kinase |
| *YAP1* | Yes1 associated transcriptional regulator |
| *YY1* | YY1 transcription factor |
| *ZEB1* | Zinc finger E-box binding homeobox 1 |
| *ZEB2* | Zinc finger E-box binding homeobox 2 |
